# Classification of Liver Trauma

**DOI:** 10.1155/1996/58383

**Published:** 1996

**Authors:** Sandro B. Rizoli, Frederick D. Brenneman, Sherif S. Hanna, Kamyar Kahnamoui

**Affiliations:** Department of Surgery and the Trauma Program Sunnybrook Health Science Centre University of Toronto Toronto Ontario Canada

## Abstract

The classification of liver injuries is important for clinical practice, clinical research and quality assurance activities. The Organ Injury Scaling (OIS) Committee of the American Association for the Surgery of Trauma proposed the OIS for liver trauma in 1989. The purpose ofthe present study was to apply this scale to a cohort ofliver trauma patients managed at a single Canadian trauma centre from January 1987 to June 1992.170 study patients were identified and reviewed. The mean age was 30, with 69% male and a mean ISS of 33.90% had a blunt mechanism ofinjury. The 170 patients were categorized into the 60IS grades ofliver injury. The number of units of blood transfused, the magnitude of the operative treatment required, the liver-related complications and the liver-related mortality correlated well with the OIS grade. The OIS grade was unable to predict the need for laparotomy or the length of stay in hospital. We conclude that the OIS is a useful, practical and important tool for the categorization of liver injuries, and it may prove to be the universally accepted classification scheme in liver trauma.


*HPB Surgery*, 1996, Vol.9, pp.235–238
Reprints available directly from the publisher
Photocopying permitted by license only

© 1996 OPA (Overseas Publishers Association)
Amsterdam B.V. Published in The Netherlands
by Harwood Academic Publishers GmbH
Printed in Malaysia

### Classification of Liver Trauma

SANDRO B. RIZOLI, FREDERICK D. BRENNEMAN, SHERIF S. HANNA, and KAMYAR KAHNAMOUI

Department of Surgery and the Trauma Program, Sunnybrook Health Science Centre, University of Toronto, Toronto, Ontario, Canada

*(Received 30 June 1994)*

The classification of liver injuries is important for clinical practice, clinical research and quality assurance activities. The Organ Injury Scaling (OIS) Committee of the American Association for the Surgery of Trauma proposed the OIS for liver trauma in 1989. The purpose of the present study was to apply this scale to a cohort of liver trauma patients managed at a single Canadian trauma centre from January 1987 to June 1992. 170 study patients were identified and reviewed. The mean age was 30, with 69% male and a mean ISS of 33. 90% had a blunt mechanism of injury. The 170 patients were categorized into the 6 OIS grades of liver injury. The number of units of blood transfused, the magnitude of the operative treatment required, the liver-related complications and the liver-related mortality correlated well with the OIS grade. The OIS grade was unable to predict the need for laparotomy or the length of stay in hospital. We conclude that the OIS is a useful, practical and important tool for the categorization of liver injuries, and it may prove to be the universally accepted classification scheme in liver trauma.

KEY WORDS: Liver injury trauma classification

### INTRODUCTION

The standardized classification of all traumatic injuries is an important goal in trauma care. However, by 1989 a number of different classifications to categorize liver injuries were in existence[1–7]. The use of these different scales created difficulty when results between clinical research studies or institutional quality assurance reviews were compared[6]. In 1989 a multidisciplinary committee appointed by the American Association for the Surgery of Trauma published the Organ Injury Scale (OIS) for liver injuries[8]. This scale, derived by the consensus of a panel of experts, was based on an existing classification scheme proposed by Moore et al. in 1984[5]. The OIS was promptly adopted by the trauma community, a group looking for a standardized classification of liver injuries.

A review of the English literature from January 1989 to May 1994 revealed that although many authors report their results using the OIS for liver trauma, there is a lack of studies validating this scale[6,9,10,11]. Curiously, one third of the manuscripts still use the Moore classification published in 1984[12].

The purpose of this study was to test the strength of the OIS classification in a group of trauma patients with liver injuries treated at a single Canadian trauma centre.

### MATERIALS AND METHODS

All patients with a traumatic liver injury admitted to Sunnybrook Health Science Centre (SHSC) from January 1987 to June 1992 were retrospectively reviewed.

Demographic data including each patient's age, sex, mechanism of injury, Injury Severity Score (ISS) and associated injuries was recorded. The description of the liver injury, based on either the operative report or the abdominal computed tomography (CT) scan in those patients who did not undergo surgery, was used to classify each hepatic injury according to the OIS as outlined in Table 1.

*Correspondence to*: Dr. F. Brenneman, Sunnybrook Health Science Centre, 2075 Bayview Avenue, Rm. H-170, North York, Ontario, Canada, M4N 3M5, Tel (416) 480-4232 Fax (416) 480-5815

**Table 1** Organ Injury scaling (OIS)[8]Committee's Classification of Liver Trauma

| *Grade* | | *Injury Description* |
|---|---|---|
| I | Hematoma: | Subcapsular, nonexpanding, < 10% surface area |
| | Laceration: | capsular tear, nonbleeding, < 1 cm deep |
| II | Hematoma: | subcapsular, nonexpanding, 10–50% surface area; intraparenchymal, nonexpanding, < 2 cm in diameter |
| | Laceration: | capsular tear, active bleeding; 1–3 cm deep, < 10 cm in length |
| III | Hematoma: | subcapsular, > 50% surface area or expanding; ruptured subcapsular hematoma with active bleeding; intra parenchymal hematoma > 2 cm or expanding |
| | Laceration: | > 3 cm deep |
| IV | Hematoma: | ruptured intraparenchymal hematoma with active bleeding |
| | Laceration: | parenchymal disruption involving 25–50% of hepatic lobe |
| V | Laceration: | parenchymal disruption involving > 50% of hepatic lobe |
| VI | Vascular: | juxtahepatic venous injury; ie. retrohepatic vena cava/major hepatic veins hepatic avulsion |

Specific management of the liver injury, either operative or non-operative, was categorized as minor or major based on previous publications[3,5,7]. These treatment categories are found in Table 2. The cause-specific mortality rate of this cohort of patients was determined. Deaths were attributed either to the liver injury (liver hemorrhage or sepsis) or to some other cause. Similarly, complications were categorized as either related or unrelated to the liver injury.

The number of units of blood transfused from the time of admission to either death or discharge from the critical care unit was noted for all patients. Although blood transfusion requirements are also determined by non-hepatic injuries, the need for transfusion increases almost exponentially with the severity of the hepatic trauma. As such, the number of blood transfusions has been used as a measure of the severity of liver injury in previous studies[4].

### RESULTS

One hundred and seventy patients with a traumatic liver injury were included in this study. The majority were male (69%) with a mean age of 30 years (range 12 to 77 years). Ninety per cent of the patients were victims of blunt trauma, most often a motor vehicle crash (69%). Only 17 patients (10%) had liver injuries from penetrating trauma. The mean ISS was 33.

**Table 2** Surgical treatment required for liver injury

| | | |
|---|---|---|
| Minor Surgical Treatment | 1 | no treatment |
| | 2 | temporary intra-operative packing, electrocautery, argon beam coagulator |
| | 3 | topical hemostatic agents |
| Major Surgical Treatment | 4 | deep liver suture |
| | 5 | Pringle manoeuvre |
| | 6 | resectional debridement, segmentectomy, hepatic lobectomy |
| | 7 | Inferior Vena Cava shunt |
| | 8 | peri-hepatic packing |

Table 3 contains all 170 patients grouped according to the OIS grade of liver injury. Nineteen patients (11%) were treated non-operatively and 151 underwent exploratory laparotomy. Of those undergoing surgery, 89 (59%) required minor surgical treatment for the liver injury (Table 2), with 35 of these patients needing no specific treatment since the liver hemorrhage had stopped spontaneously. Sixty-two patients (37%) required major surgical treatment of the liver injury. Eight patients (5%) underwent some form of hepatic resection, while caval shunting was used in 2 patients, both non‘survivors. Deep liver sutures were used in 41 patients (24%).

Twenty-five patients (15%) developed at least one complication directly related to the hepatic injury (Table 3 and Table 4). Overall, 43 of the 170 patients died, 10 (6%) as a direct consequence of the liver injury. Hemorrhage from the liver caused 7 of these deaths, all less than 48 hours after admission, while 3 patients died of liver-related sepsis, 6, 9 and 29 days respectivey after hospital admission.

Twenty-one patients (12%) did not require a blood transfusion, while the remaining 149 (88%) consumed from 1 to 95 units of packed red blood cells (mean of 13 units per patient) from the time of hospital admission to either death or discharge from the critical care unit.

### DISCUSSION

The principles of a good classification scheme for liver trauma include accurate injury descriptions, prognostic value for the prediction of outcomes, easy duplication to allow for comparisons in clinical research and, finally, simplicity and practicality for everyday use.

The OIS classification system utilizes the most accurate method of injury assessment in each case of liver trauma, either autopsy, laparotomy or radiographic investigation. The OIS was proposed to facilitate clinical research since it provides a detailed, yet simple

**Table 3** Characteristics of liver trauma study patients grouped according to OIS classification of liver injury

| *OIS Grade* | *OIS I* | *OIS II* | *OIS III* | *OIS IV* | *OIS V* | *OIS VI* |
|---|---|---|---|---|---|---|
| *Number of patients per group* | *40* | *48* | *43* | *19* | *20* | *0* |
| *Patient Characteristics:* | | | | | | *Totals* |
| Age (mean) | 35 | 28 | 31 | 26 | 25 | 30 |
| Sex: number of males (%) | 28 (70) | 37 (77) | 24 (56) | 13 (68) | 15 (75) | 117 (69) |
| ISS (mean) | 32 | 34 | 27 | 35 | 40 | 33 |
| Blunt mechanism of injury (%) | 98% | 90% | 84% | 90% | 90% | 90% |
| Mean length of stay in hospital (days) | 29 | 34 | 30 | 37 | 17 | 30 |
| Number having a laparotomy (%) | 35 (88) | 41 (85) | 38 (88) | 19 (100) | 18 (90) | 151 (89) |
| Units of pRBC transfused (mean) | 7.7 | 11.7 | 11.5 | 20.2 | 22.4 | 13.0 |
| Number requiring major treatment of liver injury (%) | 1 (3) | 9 (19) | 24 (56) | 14 (74) | 14 (70) | 62 (36) |
| Liver-related complications (%) | 0 (0) | 4 (8) | 7 (16) | 8 (42) | 6 (30) | 25 (15) |
| Number of deaths due to liver trauma | 0 | 0 | 0 | 3 | 7 | 10 |

OIS = Organ Injury Scale
ISS = Injury Severity Score
pRBC = packed red blood cells

**Table 4** Liver-Related Complications

| *Complications* | *Number of Patients* |
|---|---|
| Abscess (subphrenic, subhepatic or intrahepatic) | 16 |
| Bile leak | 8 |
| Jaundice | 7 |
| Re-bleeding | 5 |

description of each specific liver injury. The relatively uncomplicated nature of this classification scheme makes it practical for clinical use, thus improving documentation of injuries and enabling the comparison of injuries and results between different institutions. However, as with all subjective scales, it is open to some degree of interobserver variability, although the amount has never been formally quantified.

The categorization of the 170 study patients with liver trauma according to the OIS grading scheme yielded a relatively even distribution, except for lower frequencies in the higher (more severe injury) grades. No patient was found to have an OIS grade 6 injury. This is to be expected, since traumatic hepatic avulsion is an uncommon event.

The demographic characteristics of the study patients, including their age, sex and mechanism of injury were reasonably constant between different OIS groups (Table 3). The majority of trauma patients admitted to Sunnybrook Health Science Centre are victims of blunt trauma resulting in multi-system injuries. Over half of the patients are initially seen at a referring hospital and triaged prior to transfer, resulting in a skewed population of trauma victims with severe injuries. The mean ISS for the patients in this study was 33, reflecting the degree of injury severity in extraabdominal body regions. In calculating the ISS, the 3 highest Abbreviated Injury Scale (AIS) scores from different body regions are used with equal weighting[13,14]. As such, liver injuries contribute only one factor to the ISS calculation. Since many of these patients suffered multi-system trauma, the mean ISS of each OIS group was similar among study patients, regardless of the specific OIS grade assignment.

The indications for exploratory laparotomy in trauma go beyond single organ injury, since many patients have multiple abdominal injuries. Many of the patients in this series with minor liver trauma had a severe associated intra-abdominal injury requiring surgical management. As such, the proportion of patients undergoing a laparotomy in each OIS grade was similar. However, when considering the proportion of patients in each grade requiring major treatment for the liver injury, there was a predictable increase with increasing OIS grade. In a similar fashion, blood transfusion requirements were greater with higher OIS grade liver injuries. Further, the number of patients with liver-related complications and the number of liver-related deaths increased as the OIS grade of liver injury increased (Table 3). The OIS classification appears to be able to perform well in these various categories relating to clinical management.

However, the OIS does not appear to be able to predict length of hospital stay. The OIS grade with the shortest mean length of hospital stay was Grade V. This occurred because 35% of the patients in this group died early after injury due to liver hemorrhage, thereby lowering the mean length of stay for this subgroup of liver trauma patients. On the other hand, patients with mild liver injuries had such severe associated injuries that the mean length of hospital stay was close to the overall mean for the entire study group, 29 and 30 days respectively (Table 3). It is clear that OIS grading of liver injuries is a poor predictor of the length of stay in hospital.

None of the current classification systems of liver trauma are all-inclusive. Anatomic scores, such as the OIS, describe only one facet of the injured patient[2], not taking into account other variables such as age, preexisting disease and associated injuries, all of which affect outcomes[15]. However, the OIS has advantages over other existing liver injury scales. The 1984 Moore classification[5], the foundation of the OIS, is limited in use due to the restriction to intra-operative observations. In a time when CT diagnoses of liver injury are not uncommon, this diagnostic modality should be reflected in the working classification of such injuries. The OIS has made this major adjustment, such that a liver injury can now be classified even though the diagnosis and treatment may be accomplished nonoperatively.

The Buechter classification system for liver injuries, proposed in 1990[1], has not gained wide acceptance. This scale contains only 3 categories, and the patients tend to cluster into grade I injuries. If all patients in the present study were classified according to the Buechter scale, 76% would fall into grade I. This lack of precision in categorizing liver injuries limits the usefulness of this scale.

The OIS classification of liver injuries has proven to be useful and has gained acceptance in the trauma community[4,6]. It facilitates the documentation of liver injuries for the purposes of clinical research, allows case-mix comparisons between institutions, and contributes to the development of standardized principles of liver trauma management. We believe that the OIS should become the universally accepted classification scheme for categorizing liver injuries.

### REFERENCES

1. Buechter, K.J., Zeppa, R. and Gomez, G. (1990) The use of segmental anatomy for an operative classification of liver injuries. *Annals of Surgery* **211**: 669–675.
2. Champion, H.R., Copes, W.S., Sacco, W.J., Lawnick, M.M., Bain, L.W., Gann, D.S., Gennarelli, T., Mackenzie, E. and Schwaitzberg, S. (1990) A new characterization of injury severity. *Journal of Trauma* **30**: 539–546.
3. Hanna, S.S., Pagliarello, G., Taylor, G., Miller, H., Scarth, H.M.C. and Brenneman, F. (1991) Blunt liver trauma at Sunnybrook Medical Centre: a 13 year experience. *HPB Surgery* **4**: 49–58.
4. Croce, M.A., Fabian, T.C., Kudsk, K.A., Baum, S.L., Payne, L.W., Mangiante, E.C. and Britt, L.G. (1991) AAST organ injury scale: correlation of CT-graded liver injuries and operative findings. *Journal of Trauma* **31**: 806–812.
5. Moore, E.E. (1984) Critical decisions in the management of hepatic trauma. *American Journal of Surgery* **148**: 712–716.
6. Ochsner, M.G., Jaffin, J.H., Golocovsky, M. and Jones, R.C. (1993) Major hepatic trauma. *Surgical Clinics of North America* **73**: 337–352.
7. Patcher, H.L., Liang, H.G. and Hofstetter, S.R. (1988) Injury to the liver and biliary tract. In *Trauma*, edited by Mattox, K.L., Moore, E.E. and Feliciano, D.V., pp. 429–441. Norwalk: Appleton and Lange.
8. Moore, E.E., Shackford, S.R., Patcher, H.L., McAninch, J.W., Browner, B.D., Champion, H.R., Flint, L.M., Gennarelli, T.A., Malangoni, M.A., Ramenofsky, M.L. and Trafton, P.G. (1989) Organ Injury Scaling: spleen, liver and kidney. *Journal of Trauma* **29**: 1664–1666.
9. Durham, R.M., Buckley, J.B., Keegan, M., Fravell, S., Shapiro, M.J. and Mazuski, J. (1992) Management of blunt hepatic injuries. *American Journal of Surgery* **164**: 477–481.
10. Patcher, H.L., Spencer, F.C., Hofstetter, S.R., Liang, H.G. and Coppa, G.F. (1992) Significant trends in the treatment of hepatic trauma. *Annals of Surgery* **215**: 492–502.
11. Bynoe, R.P., Bell, R.M., Miles, W.S., Close, T.P., Ross, M.A. and Fine, J.G. (1992) Complications on nonoperative management of blunt hepatic injuries. *Journal of Trauma* **32**: 308–315.
12. Kasai, T. and Kobayashi, K. (1993) Searching for the best operative modality for severe hepatic injuries. *Surgery, Gynecology & Obstetrics* **177**: 551–555.
13. Committee on Injury Scaling, Association for the Advancement of Automotive Medicine: The Abbreviated Injury Scale: 1990 revision. Association for the Advancement of Automotive Medicine, 1990, Des Plaines, IL 60018, USA
14. Baker, S.P., O'Neill, B., Haddon, W., Long, W.B. (1974) The Injury Severity Score: a method for describing patients with multiple injuries and evaluating emergency care. *Journal of Trauma* **14**: 187–196.
15. Milzman DP, Boulanger BR, Rodrigues A, Soderstrom CA, Mitchell KA, Magnant CM. (1992) Pre-existing disease in trauma patients: a predictor of fate independent of age and Injury Severity Score. *Journal of Trauma* **32**: 236–244.

